# Association of anticardiolipin, antiphosphatidylserine, anti-β_2_ glycoprotein I, and antiphosphatidylcholine autoantibodies with canine immune thrombocytopenia

**DOI:** 10.1186/s12917-016-0727-3

**Published:** 2016-06-13

**Authors:** Yu-Chen Chen, Lang-Ming Chi, Kuan-Chih Chow, Shiow-Her Chiou, Yi-Hsin Fan, Shu-Peng Ho, Yu-Chen Hsu, Yu-Chyi Hwang, Meng-Xing Wu, Wei-Ming Lee, Shiun-Long Lin, Chau-Loong Tsang, Frank Chiahung Mao

**Affiliations:** Graduate Institute of Microbiology and Public Health, National Chung Hsing University, Taichung, 40227 Taiwan, Republic of China; Department of Medical Research and Development Linko Branch, Chang Gung Memorial Hospital, Taoyuan, 333 Taiwan, Republic of China; Molecular Medical Research Center, Chang Gung University, Taoyuan, 333 Taiwan, Republic of China; Graduate Institute of Biomedical Sciences, National Chung Hsing University, Taichung, 40227 Taiwan, Republic of China; Department of Veterinary Medicine, National Chung Hsing University, Taichung, 40227 Taiwan, Republic of China; Veterinary Medical Teaching Hospital, National Chung Hsing University, Taichung, 40227 Taiwan, Republic of China

**Keywords:** Anticardiolipin antibodies, Antiphosphatidylserine antibodies, Anti-β_2_ glycoprotein I antibodies, Antiphosphatidylcholine antibodies, Antiphospholipid antibodies, Immune thrombocytopenia, Antiplatelet antibodies

## Abstract

**Background:**

In humans, the presence of antiphospholipid antibodies (aPL) is frequently found in immune thrombocytopenia. The present study investigated whether aPL and any aPL subtypes are associated with canine thrombocytopenia, in particular, immune-mediated thrombocytopenia (immune thrombocytopenia) that usually manifests with severe thrombocytopenia.

**Results:**

Sera were collected from 64 outpatient dogs with thrombocytopenia (Group I, platelet count 0 – 80 × 10^3^/uL), and 38 of which having severe thrombocytopenia (platelet count < 30 × 10^3^/uL) were further divided into subgroups based on the presence of positive antiplatelet antibodies (aPLT) (subgroup I_A_, immune thrombocytopenia, *n* =20) or the absence of aPLT (subgroup I_B_, severe thrombocytopenia negative for aPLT, *n* =18). In addition, sera of 30 outpatient dogs without thrombocytopenia (Group II), and 80 healthy dogs (Group III) were analyzed for comparison. Indirect ELISAs were performed to compare serum levels of aPL subtypes, including anticardiolipin antibodies (aCL), antiphosphatidylserine antibodies (aPS), antiphosphatidylcholine (aPC), and anti-β_2_ glycoprotein I antibodies (aβ_2_GPI), and antiphosphatidylinositol antibodies (aPI), among different groups or subgroups of dogs. Among outpatient dogs, aCL, being highly prevalent in outpatient dogs with thrombocytopenia (63/64, 98 %), is an important risk factor for thrombocytopenia (with a high relative risk of 8.3), immune thrombocytopenia (relative risk 5.3), or severe thrombocytopenia negative for aPLT (relative risk ∞, odds ratio 19). In addition, aPS is a risk factor for immune thrombocytopenia or severe thrombocytopenia negative for aPLT (moderate relative risks around 2), whereas aPC and aβ_2_GPI are risk factors for immune thrombocytopenia (relative risks around 2).

**Conclusions:**

Of all the aPL subtypes tested here, aCL is highly associated with canine thrombocytopenia, including immune thrombocytopenia, severe thrombocytopenia negative for aPLT, and less severe thrombocytopenia. Furthermore, aPS is moderately associated with both canine immune thrombocytopenia and severe thrombocytopenia negative for aPLT, whereas aβ_2_GPI, and aPC are moderately relevant to canine immune thrombocytopenia. In contrast, aPI is not significantly associated with canine immune thrombocytopenia.

**Electronic supplementary material:**

The online version of this article (doi:10.1186/s12917-016-0727-3) contains supplementary material, which is available to authorized users.

## Background

In humans, the presence of antiphospholipid antibodies (aPL) is a diagnostic criterion for the antiphospholipid syndrome (APS), which manifests with thrombosis or recurrent fetal loss in women and is often accompanied with thrombocytopenia [1, 2]. APS in the absence of other related autoimmune diseases is referred to as primary APS. Besides being present in primary APS, the aPL autoantibodies are often found in patients with other autoimmune diseases, especially systemic lupus erythematosus (SLE) and immune thrombocytopenia (immune-mediated thrombocytopenia) [1, 3]. The aPL autoantibodies in humans have been recognized as lupus anticoagulants (LA), anticardiolipin (aCL), antiphosphatidylinositol (aPI), antiphosphatidylserine (aPS), antiphosphatidic acid (aPA), antiphosphatidylglycine (aPG), antiphosphatidylcholine (aPC), and anti-β_2_ glycoprotein I antibodies (aβ_2_GPI) [4, 5]. Among them, aCL and aβ_2_GPI detected by ELISA, together with LA determined by screening assays, are now required as one or more of the three laboratory criteria, along with one clinical manifestation (either vascular thrombosis or recurrent abortion in women), for the diagnosis of APS in humans [2]. Alternatively, several reports have suggested that APhL antibody ELISAs, which detect antibodies against a mixture of noncardiolipin antigens (anti-noncardiolipin phospholipids antibodies, aPhL), can be used for human APS diagnosis [6–8]. Apart from thrombosis and pregnancy morbidity, thrombocytopenia is a common manifestation in both APS (20 to 53 %) [9] and SLE patients (20 %) [10]. Although thrombocytopenia had previously been proposed to serve as a preliminary classification criterion of APS in SLE patients [11], it was not included in the later revised APS classification criteria [2]. On the other hand, a recent investigation of 35 thrombocytopenia patients with aPL found that half of them developed APS [12]. The authors of that study suggested that aPL-positive thrombocytopenia patients, along with less frequent hemolytic anemia patients, should be considered as having hematologic APS [12].

Until now, canine aPL-related studies have been limited. In 2005, the presence of aCL was detected in 33 of 63 diseased dogs by setting a cut-off value based on the sera of 134 healthy dogs, and four APS-like diseased dogs, including one dog with recurrent abortion and severe thrombocytopenia, were found to have high levels of aCL [13]. Interestingly, 22 Bernese Mountain dogs, a breed which has been shown to have a prolonged phospholipid-dependent coagulation time, presented with significantly higher levels of aCL than the controls (healthy dogs of other breeds) [14]. Nonetheless, a recent study did not correlate aPL with thrombosis or immune-mediated hemolytic anemia in dogs [15]. The involvement of aPL with thrombocytopenia, a clinical manifestation frequently occurred in APS patients, remains to be clarified in dogs.

We initiated the investigation on dogs with thrombocytopenia, in particular, immune thrombocytopenia that usually manifests with significantly lower platelet counts than thrombocytopenia of other origins [16]. The objective of this study was to explore whether any subtypes of aPL are associated with thrombocytopenia or immune thrombocytopenia in pet dogs. Indirect ELISAs were performed to measure and compare the levels and prevalence of aPhL, aβ_2_GPI, aCL, aPI, aPC, and aPS IgG antibodies in outpatient dogs with or without thrombocytopenia and in healthy dogs.

## Results

Normal platelet reference range of dogs is 200 – 500 × 10^3^/uL [17]. However, dogs with a platelet count of less than 80 × 10^3^/uL are more likely to have clinical complications [17]. In our study, serum samples were divided into three groups. Group I consisted of 64 outpatient dogs with thrombocytopenia (platelet count 0 – 80 × 10^3^/uL). Group II consisted of 30 outpatient dogs without thrombocytopenia (platelet count 250 – 500 × 10^3^/uL). To avoid any interference from other APS-related manifestations, dogs with thrombosis, pregnancy morbidity, and hemolytic anemia were not included in Group I and Group II outpatient dogs. As a control, Group III consisted of 80 healthy dogs. Group I has an average age of 5.2 years and a female to male ratio of 1.1. Group II has an average age of 5.9 years and a female to male ratio of 1.2. Group III has an average age of 2.8 and a female to male ratio of 0.7. Basic information (including breed) of three groups of dogs and clinical manifestations of outpatient dogs (Group I and Group II) are listed in the Additional file 1.

In dogs, immune thrombocytopenia often manifests with significantly lower platelet counts than other forms of thrombocytopenia [16]. Therefore, a diagnosis of presumptive immune thrombocytopenia is usually made if a dog’s platelet count falls below certain cut-off values [18]. For example, O’Marra et al. [18] have categorized thrombocytopenic dogs with platelet counts of less than 30 × 10^3^/μL and without any other underlying disease as having presumptive primary immune thrombocytopenia. Furthermore, a recent study by Iraqi et al. [19] has verified the pathological role of antiplatelet antibodies (aPLT) in the development of immune thrombocytopenia. In the present study, we selected 38 outpatient dogs with severe thrombocytopenia (platelet count < 30 × 10^3^/μL) from Group I and examined their sera for the presence of aPLT. Positive threshold for aPLT (1.5 ng/mL) was determined as 99^th^ percentile of healthy dogs (in the Group III). Group I dogs were further divided into three subgroups. Severe thrombocytopenic dogs with positive aPLT were designated as Group I_A_ (categorized as having immune thrombocytopenia) (*n* = 20), which included outpatient dogs with primary immune thrombocytopenia and those with immune thrombocytopenia secondary to other diseases. Severe thrombocytopenic dogs negative for aPLT were designated as Group I_B_ (*n* = 18). On the other hand, less severe thrombocytopenic dogs with platelet count between 30 – 80 × 10^3^/μL were designated as Group I_C_ (*n* = 26). The average level of aPLT in Group I_A_ was 3.8 ng/mL (ranging from 1.9 to 15.3 ng/mL). The results of aPLT measurement in Group I_A_, Group I_B_, and healthy control are shown in the Additional file 2.

### Expression of β_2_GPI antigen for aβ_2_GPI antibodies detection

The presence of aβ_2_GPI antibodies has been listed as one of the laboratory criteria for the diagnosis of APS [2]. For the detection of aβ_2_GPI antibodies in dogs, we cloned human β_2_GPI cDNA from HeLa cells and expressed the recombinant β_2_GPI (rβ_2_GPI). The rβ_2_GPI protein was purified by metal chelation chromatography. The purified rβ_2_GPI protein was analyzed by SDS-PAGE and Western blot, and the identity of rβ_2_GPI was verified by mass spectrometry (Additional file 3).

### Dog IgG and human aPhL IgG standard curves

**Fig. 1:**
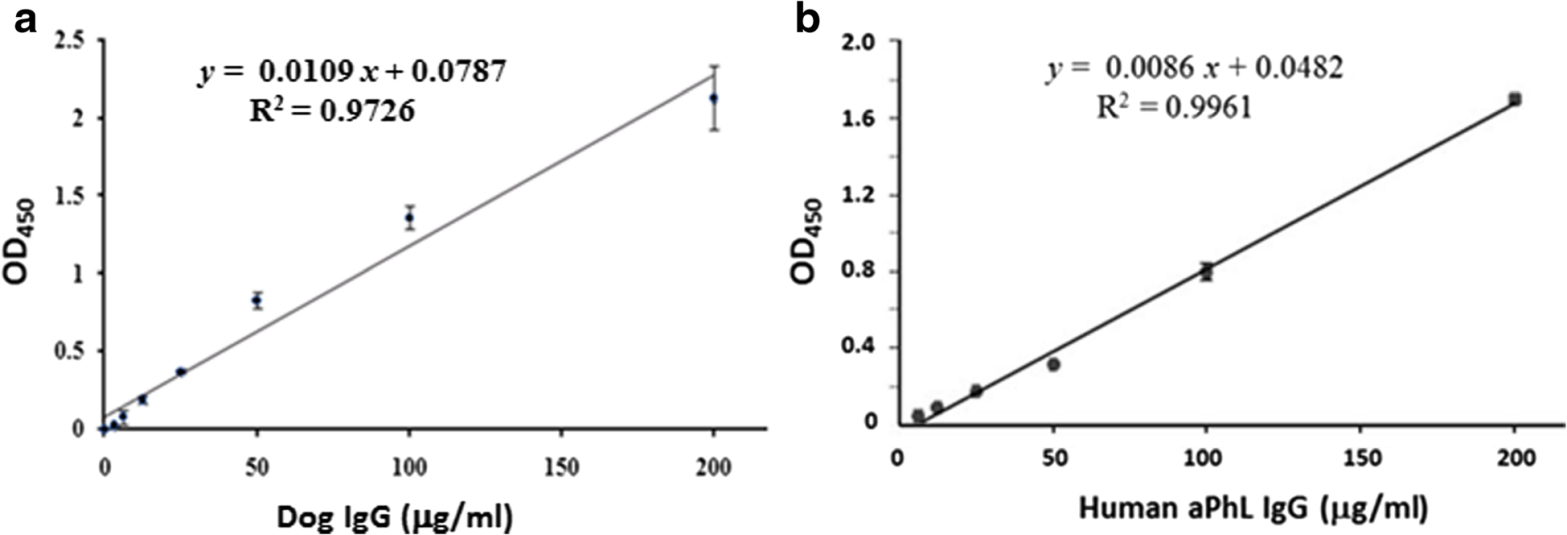
Dog IgG and human aPhL IgG standard curves. A sandwich ELISA was performed to establish a calibration curve for dog IgG (panel **a**). Rabbit anti-dog IgG F(ab)_2_ fragment antibodies were coated onto the ELISA plate. Different concentrations (200 ng/mL, 100 ng/mL, 50 ng/mL, 25 ng/mL, 12.5 ng/mL, 6.25 ng/mL, 3.125 ng/mL) of dog IgG were added into each well. Peroxidase conjugated-rabbit anti-dog IgG Fc fragment antibody was used to detect dog IgG. OD = 0.0109 × concentration + 0.0787. R^2^ = 0.9726. To obtain a human aPhL IgG standard curve, aPhL IgG reference calibrators (200, 100, 50, 25, 12.5, and 6.25 μg/mL) available in a human APhL IgG ELISA kit were used (panel **b**). OD = 0.0086 × concentration + 0.0482. R^2^ = 0.9961. The OD (450 nm) values are shown as means ± standard error (SE)

Using commercially quantified dog IgG as standards, we performed a sandwich ELISA to establish a standard curve for dog IgG calibration. Rabbit anti-dog IgG F(ab)_2_ fragment antibodies were used as capture antibodies, and dog IgG standards (200, 100, 50, 25, 12.5, 6.25, 3.125, and 0 μg/mL) were added as calibrators. A standard ELISA curve, with an R squared value of 0.9726, was generated by using linear regression (Fig. 1a). This standard equation was applied to deduce IgG concentrations (μg/mL) of various autoantibodies in canine sera (OD = 0.0109 × concentration + 0.0787).

As a comparison, reference human aPhL IgG standards (200, 100, 50, 25, 12.5, and 6.25 μg/mL) available in a human APhL IgG ELISA kit were used. A standard ELISA curve was generated by using linear regression, with an R squared value of 0.9961 (Fig. 1b).

### Comparing serum levels of aPL subtypes in different groups of dogs

By using an indirect ELISA, a mixture of non-cardiolipin phospholipids (PhL) consisting of PS, phosphatidic acid (PA), and β_2_GPI, available from an APhL IgG HRP ELISA kit, were incubated sequentially with diluted (1:50) canine sera and with HRP conjugated-rabbit anti-dog IgG antibodies to measure aPhL in canine sera. Similarly, using rβ_2_GPI, CL, PI, PC, and PS as the coated antigens, we added canine sera (1:50) and HRP conjugated-rabbit anti-dog IgG antibodies to measure aβ_2_GPI, aCL, aPI, aPC, and aPS in canine sera by indirect ELISAs.

**Fig. 2:**
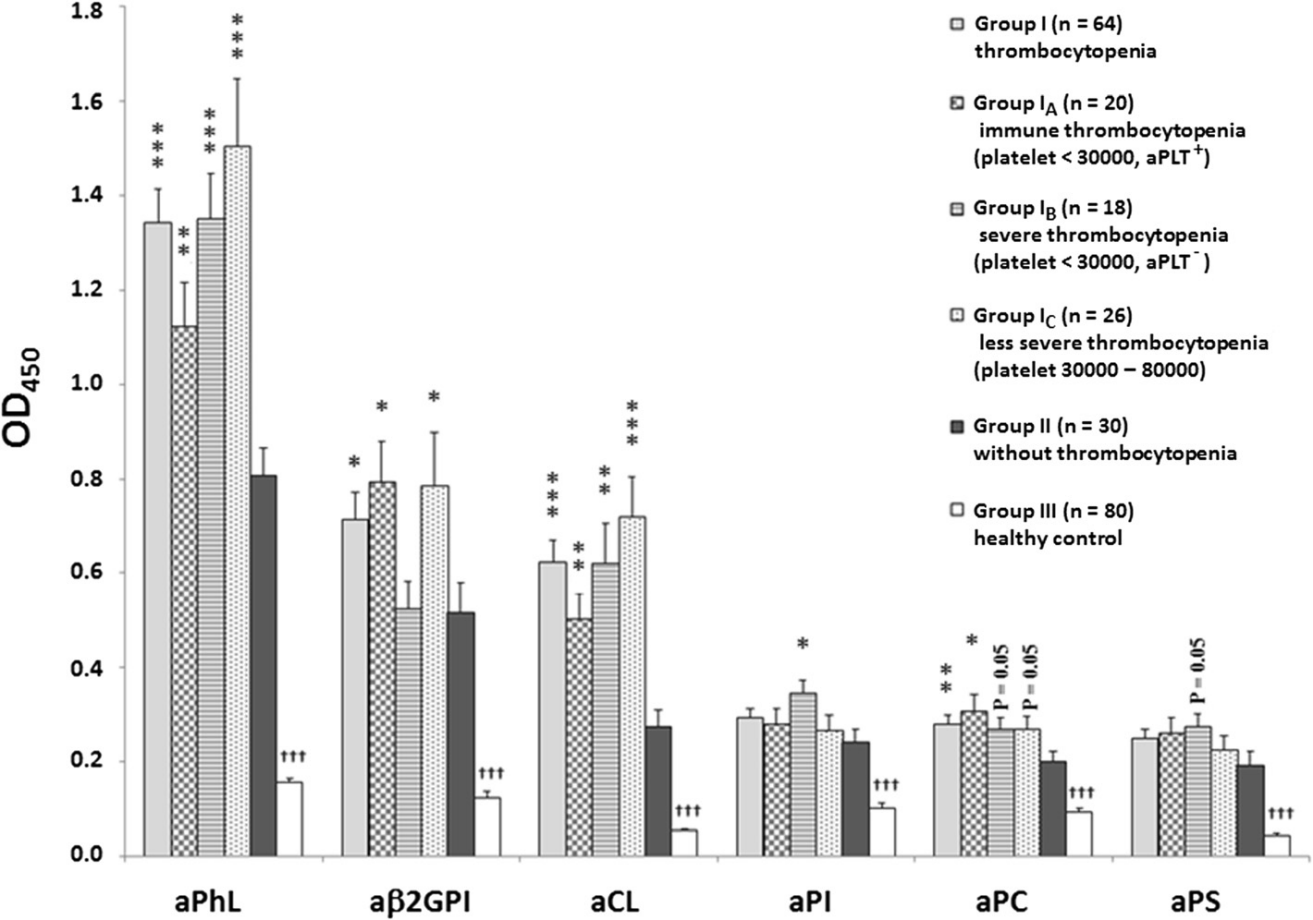
Levels of aPL subtypes determined by ELISA. The aPL subtypes in canine sera were measured. Group I: outpatient dogs with thrombocytopenia (*n* = 64). Subgroups of Group I: I_A_ (*n* = 20) (immune thrombocytopenia, aPLT^+^, platelet count < 30 × 10^3^/uL), I_B_ (*n* = 18) (severe thrombocytopenia, aPLT^-^, platelet count < 30 × 10^3^/uL), and I_C_ (*n* = 26) (less severe thrombocytopenia, platelet count 30 – 80 × 10^3^/uL). Group II: outpatient dogs without thrombocytopenia (*n* = 30). Group III: healthy dogs (*n* = 80). ^*^Group I, or subgroup I_A_, I_B_, or I_C_ is significantly higher than Group II (^***^ *P* < 0.001, ^**^ *P* < 0.01, ^*^ *P* < 0.05). ^†^Group III is significantly lower than Group I, and subgroups I_A_, I_B_, and I_C_ (^†††^ *P* < 0.001). The OD (450 nm) values are shown as means ± SE

The average ELISA OD readings for aPhL, aβ_2_GPI, aCL, aPI, aPC, and aPS obtained from outpatient dogs with thrombocytopenia (Group I), and subgroups I_A_, I_B_, and I_C_ were all significantly higher than those healthy dogs (Group III) (Fig. 2). Average OD readings for aPhL and aCL obtained from Group I and subgroups I_A_, I_B_, and I_C_ were significantly higher than those obtained from Group II (Fig. 2). In addition, compared with Group II, Group I and subgroup I_A_ had significantly higher levels of aβ_2_GPI and aPC whereas subgroup I_B_ had significantly higher level of aPI.

Based on the equation derived from the standard ELISA curve (Fig. 1), the OD readings of indirect ELISAs (Fig. 2) were converted directly into IgG levels (μg/mL or GPL) for aPhL, aβ_2_GPI, aCL, aPI, aPC, and aPS (Table 1). From high to low, average IgG levels of aPhL, aβ_2_GPI, aCL, aPI, aPC, and aPS among outpatient dogs with thrombocytopenia (subgroups I_A_, I_B_, and I_C_) were 95 – 131, 40 – 66, 39 – 60, 17 – 25, 17 –21, and 13 – 18 GPL, respectively (Table 1). On the other hand, average IgG levels of aPhL, aβ_2_GPI, aCL, aPI, aPC, and aPS in outpatient dogs without thrombocytopenia (Group II) were lower than those of Group I, but higher than those of Group III (Table 1).

**Table 1:** Average IgG levels of antiphospholipid antibodies (aPL) among different groups of dogs

aPL subtype	I_A_^a^	I_B_	I_C_	II^d^	III^e^	98^th^/99^th^percentile cut-off GPL
	(*n* = 20)	(*n* = 18)	(*n* = 26)	(*n* = 30)	(*n* = 80)	
	GPL^b^ (95 % CI)^c^	GPL (95 % CI)	GPL (95 % CI)	GPL (95 % CI)	GPL (95 % CI)	
aPhL	95.9 (79.3 – 112.5)	116.7 (99.4 – 134.0)	130.9 (105.2 – 156.6)	66.8 (56.5 – 77.0)	7.4 (5.8 – 9.1)	23.1/25.4
aβ_2_GPI	65.7 (50.6 – 80.7)	40.8 (30.2 – 51.4)	64.9 (44.6 – 85.2)	40.4 (29.0 – 51.8)	6.1 (4.0 – 8.3)	26.7/30.4
aCL	38.8 (28.8 – 48.8)	49.6 (34.3 – 65.0)	58.9 (43.8 – 73.9)	18.3 (11.9 – 24.8)	0.8 (0.2 – 1.3)	5.6/7.1
aPI	18.5 (12.6 – 24.3)	24.4 (19.2 – 29.7)	17.1 (10.8 – 23.4)	15.6 (11.4 – 19.8)	4.2 (2.4 – 5.9)	20.3/23.6
aPC	21.0 (14.5 – 27.4)	17.4 (12.7 – 22.1)	17.5 (12.6 – 22.3)	11.8 (8.4 – 15.2)	3.2 (2 – 4.4)	15.7/17.5
aPS	16.8 (11.0 – 22.5)	18.0 (12.9 – 23.0)	13.5 (8.4 – 18.6)	11.2 (6.3 – 16.2)	0.8 (0.2 – 1.4)	5.6/7.3

^a^Subgroups of Group I (thrombocytopenia): I_A_ (immune thrombocytopenia, aPLT^+^, platelet < 30 × 10^3^/uL), I_B_ (severe thrombocytopenia, aPLT^-^, platelet < 30 × 10^3^/uL), and I_C_ (less severe thrombocytopenia, platelet 30 – 80 × 10^3^/uL). ^b^Concentrations (μg/mL of IgG, GPL) were converted directly from the OD readings based on the equation shown in Fig. 1a. One GPL unit is defined as the binding activity of 1 μg/mL of IgG. ^c^95% confidence interval. ^d^Outpatient dogs without thrombocytopenia (Group II). ^e^Healthy dogs (Group III)

Because the average age of Group III healthy dogs was lower than the average ages of the outpatient dogs (Group I and Group II) (Additional file 1), we investigated whether age affected the level of antiphospholipid antibodies in healthy dogs. Group III (healthy dogs) were divided into older and younger subgroups. The average ELISA OD readings for aPhL, aβ_2_GPI, aCL, aPI, aPC, and aPS obtained from older and younger subgroups were not significantly different (Additional file 4). In addition, considering that the female to male ratio in Group III healthy dogs was lower than the ratios among the outpatient dogs (Group I and Group II), we divided Group III into female and male subgroups to explore whether gender was a factor for the antiphospholipid antibodies in healthy dogs. The average ELISA OD readings for aPhL, aβ_2_GPI, aCL, aPI, aPC, and aPS were comparable in the female and male subgroups (Additional file 4). The fact that levels of aPhL, aβ_2_GPI, aCL, aPI, aPC, and aPS were comparable between older and younger subgroups, and between female and male subgroups among healthy dogs excludes the need for setting any age- or gender-specific positive thresholds.

### Frequencies of aPL subtypes based on the 98^th^ or 99^th^ percentile cut-off values and relative risks for thrombocytopenia

The positive thresholds for aPL subtypes were determined either as the average OD values obtained from Group III healthy dogs plus two standard deviations (SD) (around 98^th^ percentile) or plus 2.33 SD (99^th^ percentile). Based on the 98^th^ or 99^th^ percentile cut-off values, the frequencies of positive aPhL, aβ_2_GPI, aCL, aPI, aPC, and aPS in outpatient dogs with thrombocytopenia (Group I and subgroups I_A_, I_B_, and I_C_) were significantly higher (*P* < 0.001) than in healthy dogs (Group III). Comparing between Group I (or subgroup I_A_, I_B_, or I_C_) and Group III, the relative risks for outpatient dogs positive for aPhL, aβ_2_GPI, aCL, aPI, aPC, aPS versus (vs.) healthy dogs negative for those aPL subtypes to develop thrombocytopenia were 73 – ∞, 7 – 30, 67 – ∞, 2 – 10, 3 – 10, and 4 – 19, respectively (Tables 2 and 3).

**Table 2:** Frequency of antiphospholipid antibodies among different groups of dogs based on the 98^th^ percentile cut-off

aPL subtype	I/I_A_/I_B_/I_C_^a^	II^b^	III^c^	I/I_A_/I_B_/I_C_	I/I_A_/I_B_/I_C_
	*n* = 64/20/18/26	*n* = 30	*n* = 80	vs. II	vs. III
	positive (%)	positive (%)	positive (%)	relative risk (odds ratio)^d, e^	relative risk (odds ratio)
aPhL	63/20/18/25 (98/100/100/96)	27 (90)	2 (3)	**2.8**/(**5.2**)/(**4.7**)/1.9	**76** ^***^/(**1287**)^***^/(**1162**)^***^/**73** ^***^
aβ_2_GPI	52/17/15/20 (81/85/83/77)	16 (53)	3 (4)	1.6^**^/**2.9** ^*^/**2.7**/1.8	**7.0** ^***^/**22** ^***^/**22** ^***^/**12** ^***^
aCL	63/19/18/26 (98/95/100/100)	20 (67)	3 (4)	**8.3** ^***^/**5.3** ^*^/(**19**)^**^/(**27**)^***^	**74** ^***^/**67** ^***^/**(819)** ^***^/**(1174)** ^***^
aPI	30/8/12/10 (47/40/67/38)	7 (23)	4 (5)	1.3^*^/1.5/**2.4** ^*^/1.4	**2.8** ^***^/**4.8** ^***^/**10** ^***^/**4.1** ^***^
aPC	38/13/12/13 (59/65/67/50)	9 (30)	4 (5)	1.4^*^/**2.3** ^*^/**2.5** ^*^/1.5	**3.5** ^***^/**9.0** ^***^/**10** ^***^/**5.2** ^***^
aPS	50/16/15/19 (78/80/83/73)	14 (47)	5 (6)	1.6^**^/**2.6** ^*^/**3.2** ^*^/1.8	**5.7** ^***^/**15** ^***^/**19** ^***^/**9.2** ^***^

^a^Subgroups of Group I (thrombocytopenia): I_A_ (immune thrombocytopenia, aPLT^+^, platelet < 30 × 10^3^/uL), I_B_ (severe thrombocytopenia, aPLT^-^, platelet < 30 × 10^3^/uL), and I_C_ (less severe thrombocytopenia, platelet 30 – 80 × 10^3^/uL). ^b^Group II: outpatient dogs without thrombocytopenia. ^c^Group III: healthy dogs. ^d^Odds ratio is shown in parenthesis if relative risk is **∞.** ^e^Value > 2 is shown in bold. ^*^Frequencies are significantly different (^*^ *P* < 0.05; ^**^ *P* < 0.01; ^***^ *P* ≤ 0.001)

**Table 3:** Frequency of antiphospholipid antibodies among different groups of dogs based on the 99^th^ percentile cut-off

aPL subtype	I/I_A_/I_B_/I_C_^a^	II^b^	III^c^	I/I_A_/I_B_/I_C_	I/I_A_/I_B_/I_C_
	*n* = 64/20/18/26	*n* = 30	*n* = 80	vs. II	vs. III
	positive (%)	positive (%)	positive (%)	relative risk (odds ratio)^d, e^	relative risk (odds ratio)
aPhL	63/20/18/25 (98/100/100/96)	26 (87)	2 (3)	**3.5** ^*^/(**6.9**)/(**6.2**)/**2.4**	**76** ^***^/(**1287**)^***^/(**1162**)^***^/**73** ^***^
aβ_2_GPI	49/17/12/20 (77/85/67/77)	16 (53)	2 (3)	1.4^*^/1.9^*^/1.4/1.8	**30** ^***^/**24** ^***^/**12** ^***^/**12** ^***^
aCL	63/19/18/26 (98/95/100/100)	20 (67)	3 (4)	**8.3** ^***^/**5.3** ^*^/(**19**)^**^/(**27**)^***^	**74** ^***^/**67** ^***^/(**819**)^***^/(**1174**)^***^
aPI	25/8/9/8 (39/40/50/31)	6 (20)	3 (4)	1.3/1.7/**2.2**/1.3	**2.2** ^***^/**5.3** ^***^/**7.1** ^***^/**3.8** ^***^
aPC	35/12/11/12 (55/60/61/46)	9 (30)	4 (5)	1.3^*^/**2.0** ^*^/**2.2**/1.4	**3.2** ^***^/**7.8** ^***^/**8.7** ^***^/**4.8** ^***^
aPS	45/16/14/15 (70/80/78/58)	13 (43)	4 (5)	1.4^*^/**2.9** ^*^/**2.7** ^*^/1.3	**4.5** ^***^/**16** ^***^/**15** ^***^/**6.2** ^***^

^a^Subgroups of Group I (thrombocytopenia): I_A_ (immune thrombocytopenia, aPLT^+^, platelet < 30 × 10^3^/uL), I_B_ (severe thrombocytopenia, aPLT^-^, platelet < 30 × 10^3^/uL), and I_C_ (less severe thrombocytopenia, platelet 30 – 80 × 10^3^/uL). ^b^Group II: outpatient dogs without thrombocytopenia. ^c^Group III: healthy dogs. ^d^Odds ratio is shown in parenthesis if relative risk is **∞.** ^e^Value > 2 is shown in bold. ^*^Frequencies are significantly different (^*^ *P* < 0.05; ^**^ *P* < 0.01; ^***^ *P* < 0.001)

Among outpatient dogs, the positive frequency of aCL (98^th^ or 99^th^ percentile cut-off) was significantly higher in Group I (98 %) and subgroup I_A_ (95 %), I_B_ (100 %), and I_C_ (100 %) than in outpatient dogs without thrombocytopenia (Group II) (Tables 2 and 3). The relative risks of aCL positive outpatient dogs (vs. aCL negative outpatient dogs) for having thrombocytopenia (Group I), immune thrombocytopenia with aPLT (subgroup I_A_), severe thrombocytopenia without aPLT (I_B_), and less severe thrombocytopenia (I_C_) were 8.3, 5.3, ∞ (odds ratio 19) and ∞ (odds ratio 27), respectively. In addition to aCL, significantly higher positive frequencies of aβ_2_GPI (85 %), aPC (65 % or 60 %), and aPS (80 %) were observed in Group I_A_ than those observed in Group II, with relative risks around 2 (1.9 – 2.9). In comparison with Group II, the positive frequency of aPS (83 % or 78 %) was significantly higher in Group I_B_, with a relative risk of 3.2 or 2.7 (Tables 2 and 3).

## Discussion

In this study, we used indirect ELISAs to measure and compare serum IgG levels of aPL subtypes (aPhL, aβ_2_GPI, aCL, aPI, aPC, and aPS) in dogs with or without thrombocytopenia and in healthy dogs. Using a secondary antibody to amplify signal, indirect ELISA is more sensitive than direct ELISA [20]. In addition, indirect ELISA allows us to use one common conjugated secondary antibody to measure different aPL subtypes.

At this point, reference dog sera are not available nor have cut-off values been established for canine aPL subtypes other than aCL. In an earlier report, Papini et al. [13] estimated canine aCL levels based on an equation obtained from measuring human reference sera and determined the cut-off value as mean plus 2 SD. In our study, we measured levels of aPL subtypes based on a standard curve established for dog IgG calibration (Fig. 1a), which appeared comparable to that generated by using reference human aPhL IgG standards (Fig. 1b). In humans, ELISA cut-off values for aCL have been diversely determined as mean plus 2 SD (around 98^th^ percentile) or 3 SD or 5 SD, or at the 95^th^ or 99^th^ percentiles [21–26]. In our present study, we assessed and compared the association of aPL subtypes with thrombocytopenia based on the 98^th^ percentile (mean of healthy dogs plus 2 SD) and 99^th^ percentile (mean plus 2.33 SD) cut-off values. Based on these cut-off values, canine sera determined as aPhL positive could compete with human reference serum (available in the APhL IgG HRP ELISA kit) for PhL (non-cardiolipin phospholipids) binding (Additional file 5), indicating that our ELISA is reliable.

We mainly investigated aPL subtypes on clinically relevant thrombocytopenic outpatient dogs (Group I) that have much lower platelet count (0 – 80 × 10^3^/uL) than Group II (250 – 500 × 10^3^/uL). This approach reduces the chance of overlapping grouping. We further divided thrombocytopenic dogs into three subgroups to explore the relevance of aPL subtypes to the severity of the disease (Group I_A_/Group I_B_ vs. Group I_C_) and to the presence or absence of aPLT (Group I_A_ vs. Group I_B_). Frequencies of aPC, and aPS appeared higher in severe thrombocytopenic dogs (Group I_A_ and Group I_B_) than in less severe thrombocytopenic dogs (Group I_C_) whereas frequencies of aCL and aβ_2_GPI were higher within thrombocytopenic outpatient dogs than in those without thrombocytopenia (Tables 2 and 3). In contrast, aPhL frequencies are comparably high among outpatient dogs (Tables 2 and 3). Interestingly, such tendencies (regarding aCL, aβ_2_GPI, aPC, aPS, aPhL) were also observed when we compared frequencies of aPL subtypes in mild thrombocytopenic dogs (platelet count 81 – 199 × 10^3^/uL) with those in Group I (platelet count 0 – 80 × 10^3^/uL) thrombocytopenic dogs or those in Group II (platelet count 250 – 500 × 10^3^/uL) (Additional file 6, Tables 2 and 3). These results suggest that aCL and aβ_2_GPI could have potential to be markers for thrombocytopenia among outpatients dogs and the presence of aPC and aPS may help to reflect the severity of thrombocytopenia.

Among all tested aPL subtypes, aCL poses the highest relative risk in outpatient dogs for having thrombocytopenia (8.3), immune thrombocytopenia with aPLT (5.3), severe thrombocytopenia without aPLT (∞, odds ratio 19), or less severe thrombocytopenia (∞, odds ratio 27) (Tables 2 and 3). This result indicates an important role of aCL in canine thrombocytopenia, including immune thrombocytopenia and particularly in severe thrombocytopenia without aPLT and in less severe thrombocytopenia. Furthermore, based on higher relative risks, outpatient dogs present with aβ_2_GPI (relative risk 2.9 or 1.9) and aPC (relative risk 2.3 or 2.0) are around two fold more likely to have immune thrombocytopenia (with aPLT) and those with aPS are two to three fold more likely to have immune thrombocytopenia (relative risks 2.6 or 2.9) or severe thrombocytopenia without aPLT (3.2 or 2.7) (Tables 2 and 3). In the future, it would be interesting to explore whether aCL, aβ_2_GPI, aPC, and aPS poses differential risks under the presence or absence of aPLT in less severe thrombocytopenia or even mild thrombocytopenia. It is worth noting that our supplement data indicate that the levels of all the aPL subtypes examined here were not significantly different between older and younger subgroups of normal healthy dogs (Additional file 4). This observation is in agreement with that reported by Budd et al. [26] for human aCL.

Although the average IgG levels of aPhL were significantly higher (*P* < 0.001) in outpatient dogs with thrombocytopenia or immune thrombocytopenia than in those without (Fig. 2), the fact that prevalences of positive aPhL in thrombocytopenia or immune thrombocytopenic outpatient dogs and in outpatient dogs with other diseases were comparably high indicates that the presence of aPhL may thus not be applicable as a diagnostic criterion for canine thrombocytopenia, immune thrombocytopenia, or APS-related disorders. Interestingly, aPhL, recognizing a mixture of non-cardiolipin antigens, which are comprised of PS, PA, and β_2_GPI, have been reported to be specifically detected in human patients with antiphospholipid syndrome (APS) and not in other disorders [6–8]. There could be disparities between human and canine APS regarding the specificity of aPL subtypes or clinical manifestations. For example, the results of our study indicate that aPhL, preferentially detected in human APS patients, may not be specific for APS-related disorders in dogs. In particular, canine aPA that was not examined individually in our present study (as opposed to two other aPhL subtypes, aPS and aβ_2_GPI) deserves future investigation. In addition, a recent study has shown that thrombosis, which is known to be associated with aPL and is listed as one marked manifestation for the diagnosis of APS in humans [2, 27], may not be correlated well with aPL in dogs [15]. Overall, here in our study we observed a high prevalence of aPL (adding up all aPL subtypes) in outpatient dogs with thrombocytopenia (98 %, Group I), immune thrombocytopenia with positive aPLT (100 %, Group I_A_), severe thrombocytopenia without aPLT (100 %, Group I_B_). Interestingly, in the recently held 14^th^ International Antiphospholipid Congress, experts in the field discussed whether to include thrombocytopenia and several other non-criteria clinical manifestations of APS as APS classification criteria [28]. Based on data presented in related medical literature and considering that aPL related thrombocytopenia increases the risk of thrombosis, Abreu et al. [28] categorize thrombocytopenia as “recommended to be included as part of APS criteria revision” although the quality of data is low. In the future, it is imperative to carry out extensive investigations to clarify whether thrombocytopenia can be listed as one clinical manifestation of human APS or canine APS-related disorders. Follow-up surveys are needed to compare the incidence of thrombocytopenia, pregnancy morbidity, hemolytic anemia, and thrombosis among dogs with positive antiphospholipid antibodies.

## Conclusions

Of all the aPL subtypes tested here, aCL is highly associated with canine thrombocytopenia, including immune thrombocytopenia, and particularly severe thrombocytopenia negative for aPLT and less severe thrombocytopenia. Furthermore, aPS is moderately associated with both canine immune thrombocytopenia and severe thrombocytopenia negative for aPLT, whereas aβ_2_GPI, and aPC are moderately relevant to canine immune thrombocytopenia. In contrast, aPI is not significantly associated with canine immune thrombocytopenia.

## Methods

### Canine blood and platelet count

EDTA-anticoagulated blood samples (for platelet count) or sera were taken from outpatient dogs admitted to the Veterinary Medical Teaching Hospital, National Chung Hsing University (Taichung, Taiwan, ROC) or from healthy dogs (based on physical examination and/or platelet count) whose owners had volunteered to bring their dogs in for a checkup. Platelet counts were measured using a KX-21 N Hematology Analyzer (Sysmex, Kobe, Japan). Owner consents were obtained before collecting blood from dogs. Sera were stored at -20 °C.

### Detection of canine aPLT by indirect ELISAs

For the detection of aPLT in serum samples, an indirect ELISA was carried out according to the manual of the canine PA-IgG/M/A ELISA kit (Novatein Biosciences, Woburn, MA, USA). Positive threshold for aPLT was determined as mean plus 2.33 SD (99^th^ percentile cut-off value) of healthy dogs (Group III of this study). Detailed method was described in the supporting information (Additional file 2).

### β_2_GPI expression and identification

Methods for the cDNA cloning, recombinant protein expression and identification of human β_2_GPI were described in the supporting information (Additional file 3).

### ELISA for dog IgG calibration

Fifty μL per well of 50 ng/mL rabbit anti-dog IgG-F(ab)_2_ fragment antibodies (Bethyl Laboratories, Montgomery, TX, USA) in ELISA coating buffer (15 mM NaCO_3_, 35 mM NaHCO_3_ at pH 9.6) were added into a 96-well plate and incubated overnight at 4 °C. The next day, each well was fixed with 100 μL of methanol for 10 min and washed twice with phosphate-buffered saline (PBS). The plate was air-dried. Each well was incubated with 100 μL of blocking buffer for 60 min. After removing the blocking buffer, 100 μL of serial 2-fold dilutions (in PBS) of dog IgG (Sigma, St. Louis, MO, USA) were added into each well and incubated for 30 min at 37 °C. After washing three times (15 min for each wash) with 200 μL of PBS, 100 μL of horseradish peroxidase (HRP)-conjugated rabbit anti-dog IgG Fc fragment specific antibodies (Jackson ImmunoResearch, West Grove, PA, USA) (1:10000 in blocking buffer, PBS containing 3 % normal rabbit serum) were added to each well and incubated for 30 min at 37 °C. After three washes, 100 μL of 3,3’,5,5’-tetramethylbenzidine (TMB) solution was added and incubated for 30 min in the dark. Finally, 100 μL of 1 M H_2_SO_4_ was added to stop the reaction. The optical density (OD) was measured at 450 nm by using a μquant ELISA reader (BioTek, Winooski, VT, USA). Each of the concentrations above were repeated in triplicate to establish a standard ELISA curve.

### An indirect ELISA for human IgG aPhL standards

Using APhL IgG calibrators (provided in the kit) containing quantified reference human aPhL IgG, an indirect ELISA was performed according to the manual of the APhL IgG HRP ELISA kit (Louisville APL Diagnostics, Doraville, GA, USA). Briefly, each of the 96 wells was incubated for 30 min with 100 μL of pre-diluted reference human sera. After three washes, HRP conjugated anti-human IgG antibodies provided in the kit was added into each well and incubated for 30 min. Substrate solution was added and incubated for 30 min in the dark. Finally, 100 μL of stop solution was added to stop the reaction. The optical density (OD) was measured at 450 nm by using a μquant ELISA reader (BioTek).

### Detection of canine aPhL, aCL, aPI, aPC, aPS, and aβ_2_GPI IgG by indirect ELISAs

The coated phospholipid antigens in an APhL IgG HRP ELISA kit (Louisville APL Diagnostics) were used as antigens to detect aPhL in canine sera. For aCL, aPI, aPC, and aPS detections, 30 μL (50 μg/mL in ethanol) of cardiolipin (CL), phosphatidylinositol (PI), phosphatidylcholine (PC), and PS (Sigma) were coated onto each of the 96 wells by evaporation. For the detection of aβ_2_GPI IgG antibodies, 50 μL of rβ_2_GPI (1 μg/mL in ELISA coating buffer) were added into each of the 96 wells. The plate was coated and fixed with methanol. The following procedures were the same as described above for the dog IgG sandwich ELISA, except that 100 μL of diluted canine serum (1:50 in blocking buffer) were added instead of adding serial 2-fold dilutions of dog IgG.

### Sequence accession number

The sequence of human β_2_GPI cDNA isolated in this study was deposited in *Gen*Bank under accession number HM212425.

### Statistical analysis

Chi-square (χ^2^) analysis, two-sided Fisher’s exact test (for small expected frequencies), and t-test were employed, and relative risks or odds ratios were calculated to correlate aPhL, aβ_2_GPI, aCL, aPI, aPC, and aPS IgG antibodies with thrombocytopenia or immune thrombocytopenia by using Prism statistical software (version 6.0, GraphPad Software, La Jolla, CA).

## Abbreviations

aCL, anticardiolipin antibodies; aPA, antiphosphatidic acid; aPC, antiphosphatidylcholine; aPG, antiphosphatidylglycine; aPhL, anti-noncardiolipin phospholipids antibodies; aPI, antiphosphatidylinositol antibodies; aPL, antiphospholipid antibodies; aPLT, antiplatelet antibodies; aPS, antiphosphatidylserine antibodies; APS, antiphospholipid syndrome; aβ_2_GPI, anti-β_2_ glycoprotein I antibodies; LA, lupus anticoagulants; rβ_2_GPI, recombinant β_2_GPI; SLE, systemic lupus erythematosus

## Additional files

Additional file 1: Basic information of three groups of dogs. (PDF 61 kb) Additional file 2: Antiplatelet antibodies measurement. (PDF 99 kb) Additional file 3: β2GPI expression and identification. (PDF 159 kb) Additional file 4: Antiphospholipid antibodies in subgroups of healthy dogs. (PDF 24 kb) Additional file 5: Competition of canine aPhL with reference human aPhL. (PDF 51 kb) Additional file 6: Frequencies of aPL subtypes in all thrombocytopenic dogs. (PDF 47 kb)
