# Supplementary material for: Association of anticardiolipin, antiphosphatidylserine, anti-β2 glycoprotein I, and antiphosphatidylcholine autoantibodies with canine immune thrombocytopenia
Source: BMC Vet Res. 2016 Jun 13;12:106. doi: 10.1186/s12917-016-0727-3 (PMC4906605; doi:10.1186/s12917-016-0727-3)
Supplement: Additional file 1: — Basic information of three groups of dogs. (PDF 61 kb) [file 12917_2016_727_MOESM1_ESM.pdf]

Table S1. Basic information of Group I (thrombocytopenia), Group II (outpatients without thrombocytopenia), and Group III (healthy) dogs

Groups of dogs	Average age	F/M	Breed (number > 1)	Clinical manifestations (number > 1)
I (n = 64)	5.2	1.1	Maltese (4), Beagle (2), Alaskan Malamute, Corgi, Pomeranian, and mixed breed (55).	I_A[a] (n = 20): severe thrombocytopenia with antiplatelet antibody (aPLT$^+$) (20), watery diarrhea, vomiting, hepatoma, Ehrlichiosis, helminthic infection, kidney stones, depression, rhino rhea and bleeding nose, and eye disease.<br>I_B[b] (n = 18): severe thrombocytopenia without aPLT (aPLT$^-$) (18), vomiting, leukopenia, Ehrlichiosis, hepatosplenomegaly, and mild cardiomegaly.<br>I_C[c] (n = 26): thrombocytopenia (26), fever (2), vomiting, hip and elbow dislocation, fibrous epulides, shock, bloody stool, bloody diarrhea and stomatitis, parvovirus infection, cough and sneeze, encephalitis, cleidocranial dysplasia, and pyometra.
II (n = 30)	5.9	1.2 (1 neutered male)	Golden Retriever (2), Formosan Mountain (2), Tibetan Mastiff, Toy Poodle, Shih Tzu, Husky, and mixed breed (22).	Sinusitis (3), watery diarrhea (2), alopecia (2), bloody urination (2), pyometra (2), leukosis (2), vomiting (2), vomiting and bloody diarrhea, ear mite, external otitis, nephrogenic systemic fibrosis, corneal ulcer, intestinal obstruction, constipation, ovariohysterectomy, iris hernia, lymphoma, transmissible venereal tumor, itchy skin, prostatic hyperplasia, enlarged bladder, weight loss, and enlarged kidney.
III (n = 80)	2.8	0.7	Maltese (7), Schnauzer (5), Formosan Mountain (3), Shih Tzu (3), Beagle (2), Labrador (2), Golden Retriever, Cocker, Bichon, Husky, Akita, Poodle, Pekingese, Shiba, Boston terrier, Dalmatian, and mixed breed (48).	Not available.

[a]Subgroup I_A (aPLT$^+$ immune thrombocytopenia); [b]subgroup I_B (aPLT$^-$ severe thrombocytopenia); [c]subgroup I_C (less severe thrombocytopenia).
