# Supplementary material for: Association of anticardiolipin, antiphosphatidylserine, anti-β2 glycoprotein I, and antiphosphatidylcholine autoantibodies with canine immune thrombocytopenia
Source: BMC Vet Res. 2016 Jun 13;12:106. doi: 10.1186/s12917-016-0727-3 (PMC4906605; doi:10.1186/s12917-016-0727-3)
Supplement: Additional file 2: — Antiplatelet antibodies measurement. (PDF 99 kb) [file 12917_2016_727_MOESM2_ESM.pdf]

Antiplatelet antibodies measurement.

Canine antiplatelet antibodies (aPLT) measurement by indirect ELISAs. For the examination of aPLT in serum samples, an indirect ELISA was carried out according to the manual of the canine PA-IgG/M/A ELISA kit (Novatein Biosciences, Woburn, MA, USA). Briefly, 50 µL of diluted canine serum samples (diluted with sample diluent) were added into each of 96 wells. One hundred µL of HRP-conjugated antibody specific for dog IgG/IgM/IgA were mixed into each well and incubated at 37 °C for 1 hour each well. After five washes, 50 µL of chromogen A and 50 µL of chromogen B solution were added and incubated for 15 min at 37 °C in the dark. Finally, 50 µL of stop solution was added and the optical density (OD) was measured at 450 nm by using a µquant ELISA reader (BioTek). Positive threshold for aPLT was determined as mean plus 2.33 SD (99th percentile cut-off value) of 10 healthy dogs (Group III of this study). For quantification, 6 pre-diluted standards (0.5 to 16 ng/mL in 50 µL) provided in the kit were used for calibration. A standard ELISA curve, with an R squared value of 0.9949, was generated by using linear regression. This standard equation, concentration (ng/mL) = 9.4117 × OD - 0.624, was applied to deduce aPLT concentrations in canine sera.

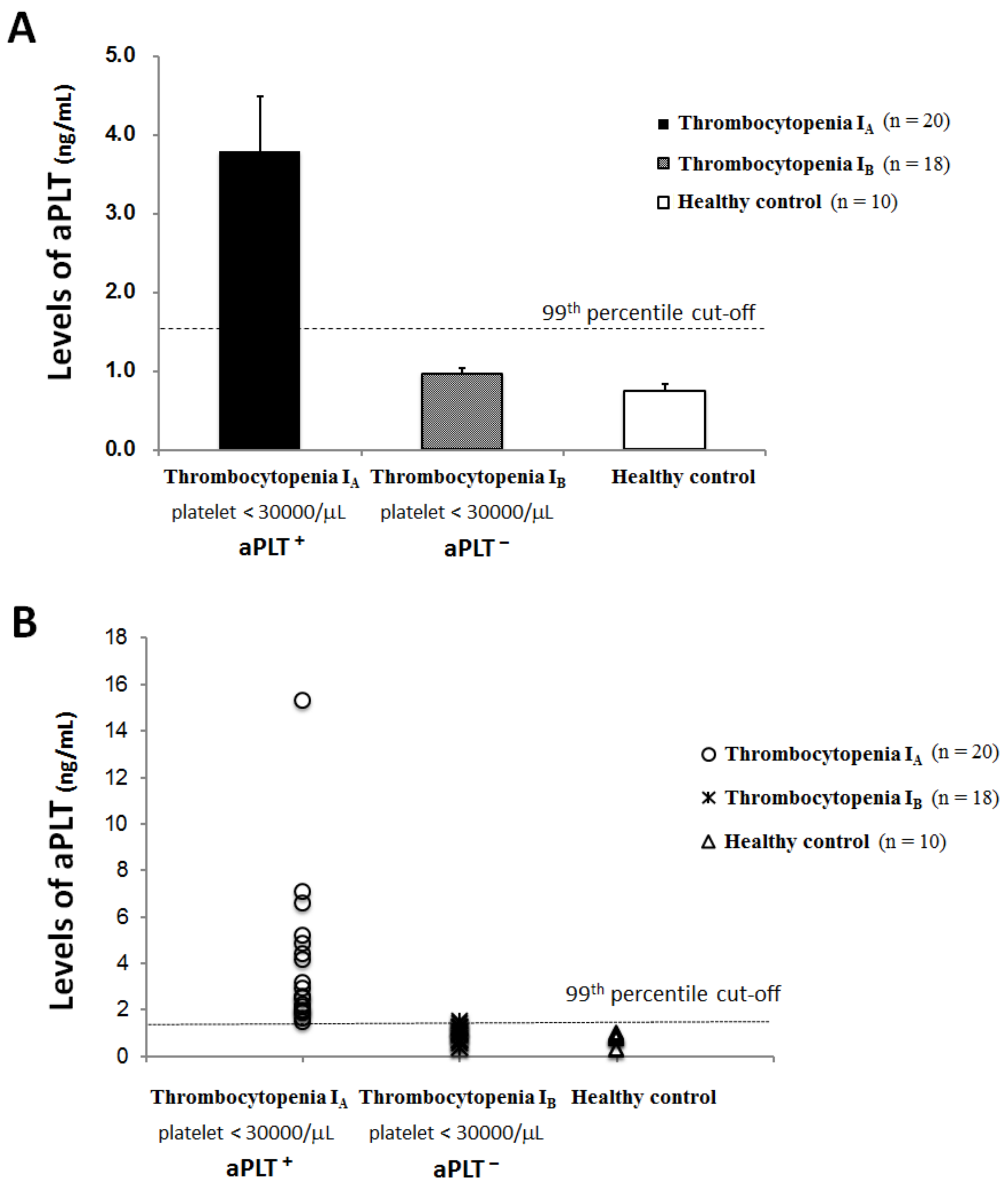

Levels of antiplatelet antibodies (aPLT). Based on a 99th percentile cut-off value, severe thrombocytopenic dogs with platelet count < 30000/μL were divided into I_A (aPLT$^+$) and I_B (aPLT$^-$) subgroups. In panel A, average levels of aPLT in subgroup I_A (severe thrombocytopenia with positive aPLT), subgroup I_B (severe thrombocytopenia with negative aPLT), and healthy dogs were presented as means ± standard error (SE). In panel B, aPLT levels of individual dogs in subgroup I_A, subgroup I_B, and healthy dogs were presented.
