# Supplementary material for: Association of anticardiolipin, antiphosphatidylserine, anti-β2 glycoprotein I, and antiphosphatidylcholine autoantibodies with canine immune thrombocytopenia
Source: BMC Vet Res. 2016 Jun 13;12:106. doi: 10.1186/s12917-016-0727-3 (PMC4906605; doi:10.1186/s12917-016-0727-3)
Supplement: Additional file 3: — β2GPI expression and identification. (PDF 159 kb) [file 12917_2016_727_MOESM3_ESM.pdf]

β_2GPI expression and identification

Molecular cloning of* β_2 glycoprotein I (*β_2GPI) cDNA. Total RNA was extracted from HeLa cells using Trizol reagent (Life Technologies, Carlsbad, CA). Primers specific for β_2GPI (5'-GGACGGACCTGTCCCAAGC-3' and 5'-TTAGCATGGCTTTACATCGGA-3') were synthesized. The cDNA encoding the mature human β_2GPI was obtained by performing reverse transcription-polymerase chain reaction (RT-PCR) and cloned into pCR2.1 vector (Life Technologies, Carlsbad, CA) to construct pCRhuβ_2GPI. Using pCRhuβ_2GPI as a template, primers 5'-TATCGAATTCGGACGGACCTGTCCCAAGC-3' and 5'-AATCGCGGCCGCTTAGCATGGCTTTACATCGGA-3' were used for the next round of PCR. The resultant β_2GPI cDNA fragment with *Eco*R I and *Not* I restriction sites (underlined in primers) was cloned into pET28 vector (Novagen, Merck KGaA, Darmstadt, Germany) to construct pEThuβ_2GPI.

Recombinant β_2GPI (rβ_2GPI) expression. *Escherichia coli* strain BL21(DE3) carrying pEThuβ_2GPI was grown and harvested after optimal induction of recombinant protein expression. Cells were lysed with a French Pressure cell press (Thermo IEC, Needham Height, MA) and rβ_2GPI protein was purified by metal chelation chromatography as described previously for the purification of histidine-tagged recombinant protein (Lin et al., 2006). The purified rβ_2GPI protein was resolved by SDS-PAGE and analyzed by Western blot (**Fig. i**).

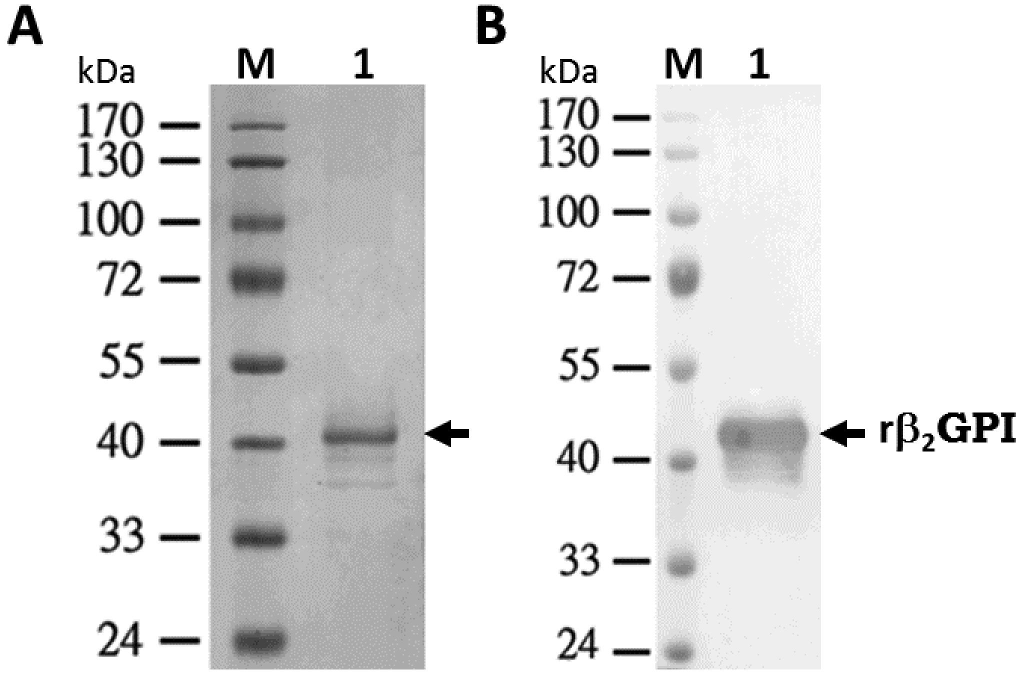

Fig. i. SDS-PAGE and Western blot analysis of the purified recombinant human β_2GPI (rβ_2GPI) protein. Recombinant protein was purified by metal chelation chromatography, resolved by 10 % SDS-PAGE (panel A, lane 1), and analyzed by Western blot (panel B, lane 1) using anti-His-tag antibody. M is prestained protein marker.

***Identification of* rβ$_2$GPI protein.** Purified rβ$_2$GPI protein was analyzed by SDS-PAGE. Gel plugs containing rβ$_2$GPI protein were collected and in-gel trypsin digestion of the protein was performed. The mass spectra were obtained by Ultraflex matrix-assisted laser desorption/ionization (MALDI) time-of-flight (TOF) mass spectrometer in reflectron mode and analyzed by using FlexAnalysis software (Bruker Daltonics, Bremen, Germany). Peptide masses obtained were searched against a comprehensive nonredundant protein sequence database (NCBInr) using the Mascot search (Perkins et al., 1999) for protein identification with the following parameters: trypsin digestion with a maximum of one missed cleavage, peptide with fixed carbamidomethylation at Cys and variable oxidation at Met, 50 ppm of peptide mass tolerance. The identity of the purified rβ$_2$GPI protein was verified by MALDI-TOF mass spectrometry (**Fig. ii**).

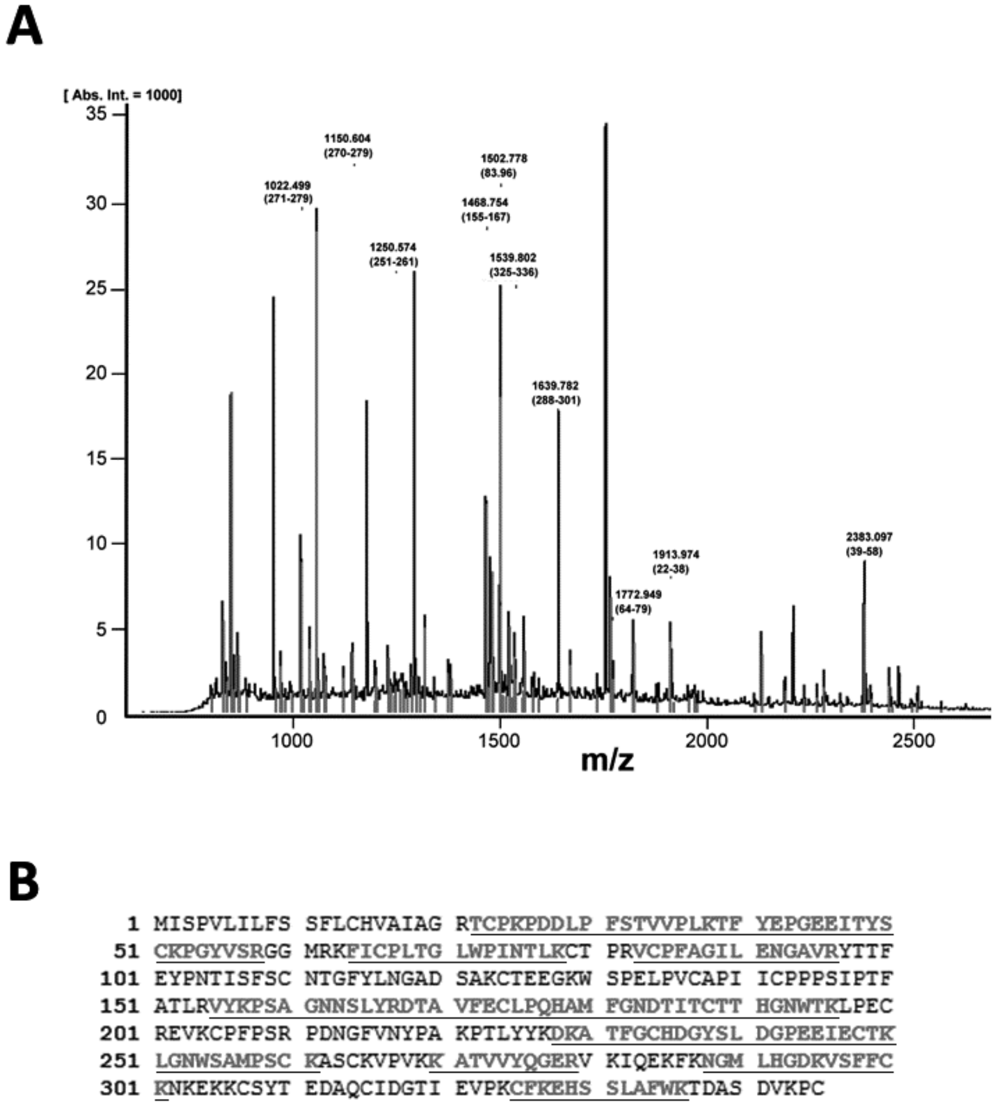

Fig. ii. Identification of recombinant human β$_2$ glycoprotein 1 (rβ$_2$GPI) by mass spectrometry. Purified rβ$_2$GPI was treated with trypsin, and the resultant peptides were analyzed by MALDI-TOF mass spectrometry. Mass spectrum (m/z) of peptides over 1000 daltons is shown (panel A). Peptide mass of human β$_2$ glycoprotein I and its amino acid position (in parenthesis) are indicated. Matched peptides are underlined (panel B).

References

Lin TY, Chan LC, Fan YH, Lin CH, Chow KC, Lin SL, Lan JL, Lin FJ, Chiou SH. Use of a recombinant protein containing major epitopes of hnRNP G to detect anti-hnRNP G antibodies in dogs with systemic lupus erythematosus. Res Vet Sci. 2006;81:335–339.

Perkins DN, Pappin DJ, Creasy DM, Cottrell JS. Probability-based protein identification by searching sequence databases using mass spectrometry data. Electrophoresis. 1999;20:3551–3567.
