# Supplementary material for: Association of anticardiolipin, antiphosphatidylserine, anti-β2 glycoprotein I, and antiphosphatidylcholine autoantibodies with canine immune thrombocytopenia
Source: BMC Vet Res. 2016 Jun 13;12:106. doi: 10.1186/s12917-016-0727-3 (PMC4906605; doi:10.1186/s12917-016-0727-3)
Supplement: Additional file 4: — Antiphospholipid antibodies in subgroups of healthy dogs. (PDF 24 kb) [file 12917_2016_727_MOESM4_ESM.pdf]

Table S2. Antiphospholipid antibodies in subgroups of healthy dogs (Group III)

	Young<br>n = 55<br>age < 4	Old<br>n = 25<br>age > 4	Y vs. O<br>*P*	Female<br>n = 32	Male<br>n = 48	F vs. M<br>*P*
age	1.33 (1.05)[a]	6.02 (2.7)	< 0.001	2.74 (2.33)	2.83 (3.09)	> 0.05
F/M	21/34	11/14		32/0	0/48	
aPhL	0.14[b] (0.08)	0.18 (0.09)	> 0.05	0.17 (0.11)	0.14 (0.07)	> 0.05
aβ_2GPI	0.12 (0.11)	0.14 (0.15)	> 0.05	0.14 (0.14)	0.11 (0.11)	> 0.05
aCL	0.05 (0.04)	0.06 (0.05)	> 0.05	0.06 (0.05)	0.05 (0.04)	> 0.05
aPI	0.10 (0.10)	0.10 (0.09)	> 0.05	0.11 (0.12)	0.09 (0.09)	> 0.05
aPC	0.10 (0.08)	0.08 (0.07)	> 0.05	0.10 (0.08)	0.09 (0.07)	> 0.05
aPS	0.05 (0.05)	0.04 (0.04)	> 0.05	0.05 (0.06)	0.04 (0.04)	> 0.05

[a]Standard deviation.

[b]The average ELISA OD readings for each subtype of antiphospholipid antibodies.
