# Supplementary material for: Association of anticardiolipin, antiphosphatidylserine, anti-β2 glycoprotein I, and antiphosphatidylcholine autoantibodies with canine immune thrombocytopenia
Source: BMC Vet Res. 2016 Jun 13;12:106. doi: 10.1186/s12917-016-0727-3 (PMC4906605; doi:10.1186/s12917-016-0727-3)
Supplement: Additional file 5: — Competition of canine aPhL with reference human aPhL. (PDF 51 kb) [file 12917_2016_727_MOESM5_ESM.pdf]

Competition of canine aPhL with reference human aPhL for antigen binding

Non-cardiolipin phospholipids (PhL) binding competition assay. The ability of canine anti-PhL antibodies (aPhL) to compete with reference human aPhL for antigen (PhL) binding was determined by ELISA. Each of the 96 wells was preincubated for 30 min with 100 µL of diluted (1:50) canine aPhL positive or aPhL negative sera, determined based on a 99th percentile cut-off value of healthy control. After three washes, each of the 96 wells was incubated for 30 min with 100 µL of 100 GPL (µg/mL) reference human aPhL, available in an APhL IgG HRP ELISA kit (Louisville APL Diagnostics). Following three washes, 100 µL of HRP conjugated anti-human IgG antibodies provided in the kit was added into each well and incubated for 30 min. Substrate solution was added and incubated for 30 min in the dark. Finally, stop solution was added and the optical density (OD) was measured at 450 nm by using a µquant ELISA reader (BioTek). The result indicates that canine sera determined as aPhL positive in this study could compete with reference human aPhL serum for PhL antigen binding. In contrast, preincubation of PhL antigen with canine aPhL negative serum did not affect antigen binding by reference human aPhL serum.

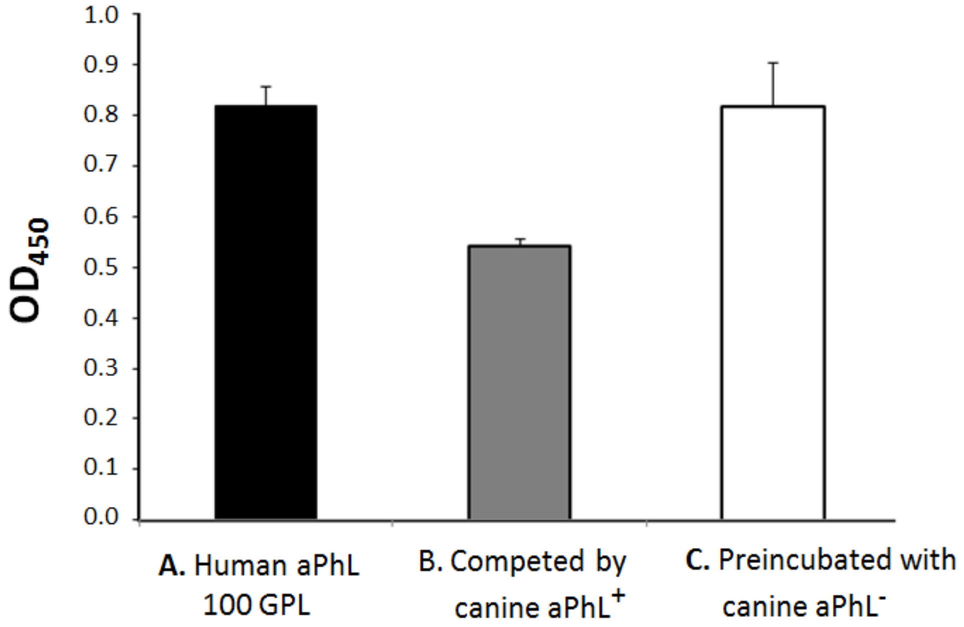

PhL binding competed by canine aPhL. PhL binding by 100 GPL of reference human aPhL was measured using an APhL IgG HRP ELISA kit (column A). For the competition assay, representative aPhL positive (column B) and aPhL negative (column C) sera were preincubated respectively with each PhL antigen-coated well prior to reference human aPhL binding. Following ELISA, the optical density (OD) was measured at 450 nm.
