# Supplementary material for: Association of anticardiolipin, antiphosphatidylserine, anti-β2 glycoprotein I, and antiphosphatidylcholine autoantibodies with canine immune thrombocytopenia
Source: BMC Vet Res. 2016 Jun 13;12:106. doi: 10.1186/s12917-016-0727-3 (PMC4906605; doi:10.1186/s12917-016-0727-3)
Supplement: Additional file 6: — Frequencies of aPL subtypes in all thrombocytopenic dogs. (PDF 47 kb) [file 12917_2016_727_MOESM6_ESM.pdf]

Table S3. Frequencies of aPL subtypes in all thrombocytopenic dogs based on the 98th percentile cut-off.

aPL subtypes	I[a] (n = 64)	eI[b] (*n* = 100)	I_{ITP}[c] (*n* = 38)	eI_m[d] (*n* = 36)
aPhL	63 (98%)	97 (97%)	38 (100%)	34 (94%)
$a\beta_2GPI$	52 (81%)	81 (81%)	32 (84%)	29 (81%)
aCL	63 (98%)	99 (99%)	37 (97%)	36 (100%)
aPI	30 (47%)	43 (43%)	20 (53%)	13 (36%)
aPC	38 (59%)	52 (52%)	25 (66%)	14 (38%)
aPS	50 (78%)	74 (74%)	31 (82%)	24 (67%)

[a]Group I: thrombocytopenia, platelet count 0 – 80×10^3/uL. [b]expanded Group I, eI: expanded thrombocytopenia, platelet count < 200×10^3/uL.

[c]Subgroup of Group I (Group I_{ITP}): presumptive immune thrombocytopenia, platelet count < 30×10^3/uL. [d]Subgroup of Group eI (Group eI_m): mild thrombocytopenia, platelet count 81 – 199 $\times 10^3$/uL.
